# Best practices for pediatric liver MRI: guidelines from members of the Society for Pediatric Radiology Magnetic Resonance and Abdominal Imaging Committees

**DOI:** 10.1007/s00247-025-06383-3

**Published:** 2025-09-10

**Authors:** Cara E. Morin, Joseph Y. Cao, Lindsay M. Griffin, Erin B. Macdonald, Morgan P. McBee, Bruce L. McHam, Scott H. Robertson, Elizabeth Tang, Gary R. Schooler

**Affiliations:** 1 Cincinnati Children’s Hospital Medical Center, 3333 Burnet Ave, Cincinnati, OH 45229 United States; 2 University of Cincinnati, Cincinnati, United States; 3 Duke University, Durham, United States; 4 Connecticut Children’s Medical Center, Hartford, United States; 5 Medical University of South Carolina, Charleston, United States; 6 Marshfield Clinic, Marshfield, United States; 7 Children’s Hospital Colorado, Aurora, United States

**Keywords:** Liver, Pediatric, SPR

## Abstract

Magnetic resonance imaging (MRI) has become an essential tool in the evaluation of pediatric liver disease. However, the unique physiological, anatomical, and behavioral characteristics of pediatric patients present distinct challenges that necessitate tailored imaging strategies. These guidelines, developed by members of the Society for Pediatric Radiology (SPR) Magnetic Resonance and Abdominal Imaging Committees, provide comprehensive recommendations for performing high-quality liver MRI in children. Drawing on multidisciplinary expertise from pediatric radiologists, MRI physicists, and technologists across diverse institutions, the document addresses key technical considerations, including field strength selection, motion mitigation, contrast agent use, and age-specific protocol optimization. These guidelines aim to standardize and elevate the quality of pediatric liver MRI, offering practical, evidence-informed recommendations to support safe, efficient, and diagnostically robust imaging across a wide range of clinical scenarios.

**Supplementary Information:**

The online version contains supplementary material available at 10.1007/s00247-025-06383-3.

## Introduction

The use of MRI to evaluate congenital and acquired liver diseases in children is the standard of care for many clinical indications, including the evaluation of biliary abnormalities [1], vascular anomalies [2], liver tumors [3], and quantitative liver imaging [4]. These diseases occur from the neonatal period to the teenage years, encompassing children of a wide spectrum of sizes and varying ability to follow commands. Many routine abdominal MR imaging sequences require breath-holds, which generate challenges in both awake and sedated/anesthetized pediatric patients. These challenges require either adapting existing sequences for use during free-breathing or utilizing sequences specifically designed to compensate for respiratory motion. Additionally, simple changes in parameters (e.g., decreasing the field of view for infants) modify many other parameters as a cascade in the background, leading to struggles with real-time image quality optimization in children. It is helpful to provide technologists with pre-optimized protocols tailored to children of different sizes and breath-holding abilities. Given the unique technical and diagnostic challenges of MR evaluation of infants and children with abdominal diseases, there has been interest in creating guideline documents for imaging pediatric patients. Herein, we provide guidelines and imaging suggestions to address common imaging challenges in children, including the standard imaging sequences obtained for the purpose of liver evaluation.

These guidelines and imaging suggestions reflect a concerted effort of members of the SPR MRI and Abdominal Imaging Committees who have specific expertise in pediatric liver imaging, clinical MRI physicists, and pediatric MRI technologists, all from children’s imaging programs of various sizes with a variety of common MRI vendor platforms. The aim of these guidelines is to provide practical guidance for the implementation and application of up-to-date MR imaging techniques in all pediatric patients. The final document was reviewed and approved by the full MRI and Abdominal Imaging Committees and ultimately approved by the SPR Board.

### Field strength considerations

Pediatric liver MRI is typically performed at 1.5 T or greater. The advantages and limitations of different field strengths are noted below.

1. Higher field strengths (e.g., 3 T) offer improved signal-to-noise ratio (SNR), enabling higher resolution and/or faster imaging.
2. Higher field strengths tend to have poorer magnetic field homogeneity, which can make chemical fat saturation difficult to achieve consistently.
  - Alternative fat suppression methods, such as STIR or Dixon techniques, are less sensitive to inhomogeneities.
3. Reduced magnetic field homogeneity also makes geometric distortions more severe, which is particularly problematic in diffusion-weighted imaging (DWI).
  - Decreasing echo spacing by increasing receiver bandwidth, using parallel imaging acceleration, or performing multi-shot diffusion imaging can reduce distortion.
4. Susceptibility artifacts are more prevalent at higher field strengths. Using shorter echo times or a higher receiver bandwidth is an effective strategy to manage these artifacts [5].
5. MRI at 1.5 T may be preferred for liver iron quantification since T2* decay is slower at 1.5 T, enabling a larger range of echo times to be employed with sufficient SNR for iron quantification. Additionally, more calibration data is available at 1.5 T than 3 T [5].
6. MRI safety concerns are typically more restrictive at higher field strengths because of the increased radiofrequency (RF) energy deposition and increased specific absorption rate (SAR). In addition, devices and implants that are MR conditional at one field strength may be unsafe at a different field strength. MRI conditionality statements should always be reviewed for each field strength used for imaging.

### Specialized imaging equipment

1. RF coils should be selected based on patient size and they should be oriented to maximize the benefits of acceleration.
  - Coil arrays with sizes closely matching the desired field of view FOV) will offer improved image SNR.
  - Dedicated pediatric coils are offered by multiple MRI vendors and third-party vendors [6].
  - Adult coil arrays with high element densities, such as cardiac arrays, head/neck vascular coils, and flexible extremity coils, are good alternatives to dedicated pediatric coils depending on patient size [7].
  - Multichannel arrays enable parallel imaging for accelerated acquisitions. Larger channel count coils offer greater potential for image acceleration. However, the advantages of greater imaging speed must be balanced with the inherent SNR loss from parallel imaging.
2. MR elastography exams require additional phase-sensitive pulse sequences, an elastography driver, and post-processing software.
3. Diffusion-weighted imaging benefits from high-performance gradients, though they are not a requirement.
4. Iron and fat quantification typically requires additional multi-echo pulse sequences and post-processing software.

### Gadolinium-based contrast agents

Pediatric liver MRI routinely uses the paramagnetic properties of gadolinium-based contrast agents (GBCAs) to enhance lesion detection and characterization. However, group I classified GBCAs add a potential risk of nephrogenic systemic fibrosis (NSF) in patients with stage 3 or worse renal failure [8]. There is also evidence of gadolinium accumulation in other tissues [9]. To reduce potential toxicity, gadolinium is chelated with other compounds, which are typically described by their relaxivity and are classified by the American College of Radiology (ACR) according to their stability.

The eight current FDA-approved GBCAs for pediatrics are listed in Table 1. GBCA safety and administration considerations are noted below.

1. Non-contrast techniques should be considered alternatives for contrast-enhanced techniques whenever possible.
2. Special consideration to minimize gadolinium dose should be based on the child’s measured weight and renal function in addition to GBCA relaxivity and stability.
  - Higher relaxivity GBCAs, such as gadopiclenol, offer the ability to use lower gadolinium dosages.
  - Macrocyclic compounds are the most stable class of GBCAs and pose the lowest risk of NSF.
3. Patients who have elevated total bilirubin and/or evidence of liver failure may have poor excretion of gadoxetate into the biliary tree, rendering MRCP evaluation limited or non-diagnostic [10]. Assessment of these factors prior to examination is recommended.

**Table 1:** Current FDA-approved gadolinium-based contrast agents approved for pediatric use

Contrast agent	Trade name	Type	T1 relaxivity in human plasma at 37 °C mM^−1^ s^−1^	T2 relaxivity in human plasma at 37 ℃, LmM^−1^ s^−1^	Clearance	ACR classification	FDA-approved ages
			(1.5 T/3 T)*	(1.5 T/3 T)			
Gadodiamide	Omniscan	Linear	4.3/4.0	5.2/5.6	Renal	Group I	≥ 2 years
		Non-ionic					
Gadopentetate dimeglumine	Magnevist	Linear	4.1/3.7	4.6/5.2	Renal	Group I	≥ 2 years
		Non-ionic					
Gadobenate dimeglumine	MultiHance	Linear	6.3/5.5	8.7/11.0	Renal (95–96%) and hepatobiliary (4–5%)	Group II	All ages
		Non-ionic					
Gadobutrol	Gadavist/ Gadovist	Macrocyclic non-ionic	5.2/5.0	6.1/7.1	Renal	Group II	All ages
Gadoteric acid	Dotarem	Macrocyclic ionic	3.6/3/5	4.3/4.9	Renal	Group II	All ages
Gadoteridol	ProHance	Macrocyclic non-ionic	4.1/3.7	5.0/5.7	Renal	Group II	All ages
Gadoxetate disodium	Eovist/	Linear	6.9/6.2	8.7/11.0	Hepatobiliary (50%) and renal (50%)	Group III	All ages
	Primovist	Ionic					
Gadopiclenol	Vueway/Elucirem	Macrocyclic non-ionic	19/9.9	34/60	Renal	Group II	≥ 2 years

Information in this table is based on the following references:

Robic C, Port M, Rousseaux O, Louguet S, Fretellier N, Catoen S, Factor C, Le Greneur S, Medina C, Bourrinet P, Raynal I, Idée JM, Corot C. Physicochemical and pharmacokinetic profiles of gadopiclenol: a new macrocyclic gadolinium chelate with high T1 relaxivity. Invest Radiol. 2019 Aug;54(8):475–484. PMCID: PMC6661244

Shen Y, Goerner FL, Snyder C, Morelli JN, Hao D, Hu D, Li X, Runge VM. T1 relaxivities of gadolinium-based magnetic resonance contrast agents in human whole blood at 1.5, 3, and 7 T. Invest Radiol. Ovid Technologies (Wolters Kluwer Health); 2015 May;50(5):330–338. PMID: 25,658,049

Rohrer M, Bauer H, Mintorovitch J, Requardt M, Weinmann HJ. Comparison of magnetic properties of MRI contrast media solutions at different magnetic field strengths. Invest Radiol. Ovid Technologies (Wolters Kluwer Health); 2005 Nov;40(11):715–724. PMID: 16,230,904

### Motion management

Pediatric MRI has unique considerations that influence motion management strategies. Coaching patients to perform breath-holds and remain still is less successful in younger pediatric populations. Consequently, prioritizing patient comfort, employing effective motion mitigation strategies, utilizing rapid imaging techniques, and incorporating respiratory gating become crucial elements in achieving diagnostic image quality. Considerations for pediatric motion management are noted below.

#### Patient comfort and preparation

1. Patient cooperation with lying still and performing breath-holds can be improved by reducing patient anxiety [11].
  - Child life specialists provide education, support, and comfort for children and their families before and during their imaging procedure.
  - Practicing breath-holds before imaging may help patients get used to the required breath-hold durations and inform the technologist about their breath-holding capabilities.
  - Decorating MRI rooms and equipment with child-friendly themes reduces anxiety.
  - Dedicated real or virtual mock MRI environments that simulate the equipment, sounds, and vibrations prepare children for what to expect during an MRI exam.
  - Audiovisual equipment captures the child’s attention during the procedure, fostering compliance.
  - Hearing protection, including ear plugs and headphones, reduces the potential for permanent hearing loss and also prevents motion induced by startling MRI sounds. Many vendors now also offer quieter gradient modes, which can be helpful to reduce anxiety.
2. For infants up to 6 months of age, feeding and bundling are effective at reducing bulk patient motion, but do not improve respiratory motion artifacts [12].
3. For patients more than 6 years old, breath-hold imaging may be possible with appropriate coaching. Breath-holds are typically restricted to 16 s or less, which constrains the achievable spatial resolution and coverage.
4. For young pediatric patients, between 6 months and 6 years old, sedation or general anesthesia may be needed to suppress patient motion to achieve diagnostically useful images. A risk–benefit analysis must be incorporated into the decision whether to use sedation or anesthesia or rely on other motion mitigation strategies. The depth and length of anesthesia should be considered, as breath-holds may require deeper anesthesia [6]

#### Motion mitigation sequences and strategies

1. The effects of periodic translational motion can be reduced by frequently sampling the center of k-space. This can be accomplished with sampling strategies that use radial blades of parallel lines (e.g., BLADE—Siemens Healthineers, PROPELLER – GE HealthCare, MultiVane XD – Philips Healthcare) or radial spokes (GRASP, StarVIBE—Siemens; Disco Star, LAVA Star – GE Healthcare; 4D Free Breathing, 3D Vane XD—Philips). Oversampling the k-space center can increase scan duration, so most clinical implementations mitigate this time penalty via parallel imaging or compressed-sensing reconstruction.
  - Through-plane motion is not improved by two-dimensional sequences that use conventional Cartesian trajectories in the slice direction [13].
2. Signal averaging can be used to reduce the conspicuity of motion artifacts in MR images.
  - Additional averages lengthen the total image acquisition time [13].
  - Phase oversampling, rather than full signal averages, can also be used to mitigate motion with less impact on imaging time [13].
3. Swapping the phase-encoding direction can confine the motion artifacts to regions that do not overlap the liver. Caution may be warranted as phase artifacts may result and mimic pathology.
4. Employing fat suppression or saturation bands on the subcutaneous fat in the anterior abdomen wall can help to reduce motion artifacts from the hyperintense peripheral fat in the moving anterior abdominal wall.

#### Rapid imaging and acceleration sequences and strategies

1. Single-shot and multi-shot imaging acquire multiple lines of k-space data for each TR, accelerating image acquisition at the expense of the caveats noted below.
  - Longer echo trains reduce contrast resolution, mix contrast weighting, and can cause T_2_-blurring [13].
  - Additional RF pulses in single- or multi-shot spin echo imaging increase SAR.
2. Parallel imaging uses multiple receive coil elements to acquire fewer phase encodes, accelerating imaging 2–fourfold depending on the FOV, receive coil design, and channel count [14].
  - There are two strategies of parallel imaging: image-based (SENSE – Siemens, ASSET – GE Healthcare, SENSE—Philips) and k-space-based (GRAPPA—Siemens, ARC – GE Healthcare). K-space-based parallel imaging typically manifests fewer artifacts for abdominal imaging, but using either parallel imaging strategy is highly recommended to reduce scan time and breath-hold duration [6].
  - CAIPIRINHA (Siemens), an extension to 3D GRAPPA, can offer additional acceleration in pediatric liver MRI [13]
3. Partial Fourier imaging (partial Fourier—Siemens, Fractional NEX—GE Healthcare, Half Scan—Philips), a technique that exploits symmetries in k-space, is useful to further reduce scan time and breath-hold duration.
  - Partial Fourier imaging offers a maximum two-fold acceleration; although practically, image acquisition is expedited by less than two-fold to preserve image quality [15].
  - Greater time savings are achievable when partial Fourier is used in the phase-encoding direction rather than the readout direction [6].
  - Further acceleration can be achieved by using partial Fourier in conjunction with parallel imaging.
4. Compressed sensing and deep learning can now be exploited on modern clinical MRI scanners to further reduce scan and breath-hold times, offering two- to five-fold acceleration [14, 16].
  - Image reconstruction with these techniques is slower, due to its iterative nature, and inherently results in image filtering.
  - Physician feedback is needed to balance acceleration and filtration.
    (i) High acceleration rates can lead to overly smooth images [6].
  - Compressed sensing and deep learning are typically used with parallel imaging to further accelerate image acquisition. A careful and informed approach is advised with the application of deep learning reconstructions in pediatric MR [17].
5. Simultaneous multi-slice (SMS) imaging expedites imaging by concurrently acquiring multiple slices (simultaneous multi-slice—Siemens, Hyperband—GE Healthcare, MultiBand SENSE—Philips)
  - Acceleration factors on the order of two-fold are achievable with SMS [13].
  - SMS is compatible with parallel imaging in-plane.

#### Respiratory synchronization

Respiratory motion can be monitored in real-time to restrict imaging to specific phases of respiration. Respiration can be monitored using external triggers (e.g., respiratory bellows) or internal triggers (e.g., navigator echoes).

1. Navigator echoes more accurately track diaphragmatic motion, capturing shallow and irregular breathing more reliably than bellows [13].
2. Respiratory gating techniques can increase scan time by 2–threefold or more, depending on the breathing pattern [13].
3. Respiratory gating using adaptive intelligence is available on some modern scanners. This allows for accurate triggering without the need for respiratory bellows or an additional navigator pulse (e.g., Philips VitalEye).

### Age-specific protocol optimization

The large variability in pediatric patient sizes and their ability to breath-hold complicates protocol optimization and makes the use of a single protocol for all patients inefficient.

1. Pediatric patients have smaller anatomical features than adults, requiring higher spatial resolution imaging that could result in unnecessarily long scan times for a one-size-fits-all FOV.
2. Pediatric patients of different ages also have differing abilities to perform a breath-hold and remain still during scanning, influencing the selection of motion mitigation strategies.
3. It is recommended to have at least two, and preferably three, pre-optimized protocols for children of different sizes and abilities to breath-hold on your scanner for technologists to choose from. Suggested protocol sets for different pediatric patient ages are listed in Table 2 along with typical FOVs.

**Table 2:** Typical motion management strategies and fields-of-view (FOV) for various age categories in pediatric liver imaging

Age	Motion management	Axial FOV (mm)	Coronal FOV (mm)
Infant/neonate (< 6 months)	Free-breathing. Feed and bundle up to 6 months	200	220
Toddler (6 months–6 years)	Free-breathing. Usually sedated	250	280
School-aged (> 6 years)	Non-sedated free-breathing exams are possible in some children	300	320
Teenager	Breath-hold coaching with motion mitigation	330	340

### General techniques

MRI of the liver is performed for a wide spectrum of focal and diffuse liver diseases in children. Typical MR protocols will include core sequences performed in most cases to evaluate liver anatomy and parenchyma, with the addition of specific sequences tailored for the indication and age and size of the patient. Here, we include basic technical considerations, diagnostic justification, special motion considerations, and other tips for common sequences used in pediatric liver evaluation (Table 3). More detailed technical parameters for consideration are included in the supplemental materials.

**Table 3:** Common sequences for pediatric liver MR

Liver anatomy and general parenchymal evaluation
Axial T2 FS (optional axial T2 non-FS, optional coronal T2)
Axial T1 in/opposed-phase or Dixon (IP/OP/F/W)
Optional: axial 2D bSSFP
Optional: axial T1 FS post-contrast
**Additional sequences for liver mass**
Axial DWI (*B* = 50, 800 s/mm^2^)
Axial T1 FS pre-contrast
Axial T1 FS dynamic post-contrast (30 s, 60 s, 3 min)
Coronal T1 FS post-contrast (3 min)
**Additional sequences for biliary imaging**
Coronal 2D thick slab T2 SS-FSE MRCP
Coronal 3D T2 MRCP
Axial/coronal T1 FS hepatobiliary-phase contrast-enhanced MRCP
**Additional sequences for quantitative evaluation**
Iron quantification: R2*/R2
Fat quantification: PDFF
Stiffness quantification: MR elastography
**Additional sequences for vascular imaging**
3D spoiled GRE (post-contrast MRA): GBCA
3D spoiled GRE (post-contrast MRA): ferumoxytol
Time-resolved MRA

### Liver anatomy and general parenchymal evaluation

The following sequences are typically included in all liver protocols.

1. Axial T2 FS (optional axial T2 non-FS; optional coronal T2)
  - Technical considerations:
    (i) FOV: The complete abdomen (the lung base to iliac crest) for axial. For optional coronal, include the liver and through as much of the pelvis as possible.
    (ii) Use an appropriate TE for respective magnet strength to ensure adequate T2-weighting; between approximately 80–100 ms should be used at 1.5 T and 70–100 ms at 3 T [18].
    (iii) Optional: axial T2 non-FS and coronal T2. Use of single-shot and avoidance of fat saturation can speed this additional sequence [12]. Orthogonal plane can supplement anatomic characterization.
  - Justification: Provides characterization of parenchyma, biliary tract, and any mass. Fat saturation during T2-weighted liver imaging highlights T2-hyperintense areas of liver parenchymal fibrosis and edema/inflammation in surrounding tissues, and differentiates between T2 hyperintensity secondary to macroscopic fat versus fluid. Often, vessels demonstrate flow-voids on T2-weighted imaging, which can suggest patency, particularly useful when post-gadoxetate sequences may not fully opacify the vessel.
  - Motion considerations: Speed of single-shot technique can mitigate motion artifacts, but T2 contrast resolution can be lower on single-shot than on traditional fast/turbo spin-echo sequences or STIR [12, 13].
  - Pitfalls and pearls/tips and tricks:
    (i) Fat saturation increases sequence duration.
    (ii) Pre-contrast STIR can provide more homogeneous fat suppression than fat-sat T2 spin-echo sequences. Note that STIR should not be used post-contrast, such as during “dead time” prior to hepatobiliary phase, as gadolinium T1 effects can alter STIR signal intensity [19].
    (iii) Fast/turbo spin-echo or single-shot T2-weighted sequences could be performed post-contrast with no substantial effects on liver signal.
    (iv) In large, obese pediatric patients and/or patients with ascites, standing wave/dielectric effect artifacts can worsen at higher magnet strengths, i.e., 3 T; consider use of < 3 T MR scanner for these patients.
2. Axial T1 in-phase/opposed-phase or Dixon
  - Technical considerations:
    (i) FOV: The complete abdomen (the lung base to iliac crest)
  - Justification: Provides anatomic detail, parenchymal T1 signal assessment, chemical shift characterization of fat/lipid and potentially iron content, and evaluation of any mass.
  - Motion considerations: Conventional 2D GRE in-/opposed-phase sequence can require multiple breath-holds; a saturation band may decrease motion artifact but does increase scan time [20]. Newer, single breath-hold and free-breathing 3D Dixon techniques are available, which can provide, in a single sequence, the in-/opposed-phase reconstructions, along with additional water-only and fat-only reconstructions [19].
  - Pitfalls and pearls/tips and tricks:
    (i) In-phase sequence is typically derived from longer TE than opposed-phase, which results in relative in-phase susceptibility signal dropout (e.g., from iron, air).
    (ii) Selection of in-phase TE shorter than opposed-phase TE should generally be avoided if possible, as relative opposed-phase signal dropout can confusingly be from either lipid or susceptibility in this scenario.
    (iii) Fat–water swapping artifacts can occur with the Dixon technique, which can impact the whole image or regions of the image. When present, all four Dixon reconstructions should be sent for evaluation. Alternatively, all four reconstructions can be sent routinely.
3. Axial bSSFP (optional)
  - Technical considerations:
    (i) FOV: The complete abdomen (the lung base to iliac crest) or entire biliary tree
    (ii) Field strength: Greater susceptibility artifact at 3 T
  - Justification: Balanced steady-state free precession sequence (using the shortest possible TR to decrease susceptibility) can be a supplemental, relatively motion-insensitive sequence for ductal and vascular characterization due to bright blood appearance, when motion limits other T2 sequences [12]. Radiofrequency pulses maintain a steady state of longitudinal and transverse magnetization, which results in a mixed contrast image based on T2/T1 ratios and leads to bright signal in both arteries, veins, and bile ducts.
  - Motion considerations: Respiratory navigation is usually needed as scan time exceeds ability to breath-hold.
  - Pitfalls and pearls/tips and tricks:
    (i) Balanced steady-state free precession can be employed for both 2D and 3D MRCP. This technique, based on gradient-echo sequences, utilizes short repetition times, resulting in brief acquisition times even less than a second per slice. While typically performed with a breath-hold technique, it can also be executed using a respiratory-gated approach.
    (ii) For vascular imaging, coronal 3D isotropic acquisition with multiplanar reformats yields the shortest scan time and T2-preparation pulses and fat saturation can be added to increase vessel conspicuity.
    (iii) SSFP is very sensitive to field inhomogeneities which can lead to extensive banding artifacts if post-surgical material is present and/or at air interfaces (such as bowel gas and lung bases when considering liver MRA).
    (iv) Flow artifact in hepatic and portal veins as well as tortuous collaterals/varices can occasionally limit assessment of the portal and systemic venous structures.
4. T1 FS post-contrast (optional): Note that post-contrast T1 FS imaging for liver MR typically entails dynamic, multi- and/or hepatobiliary phases of contrast, to maximize benefit, as detailed in indicated protocols below. A single venous phase, post gadolinium-based contrast T1 FS sequence can be pursued for general abdominal evaluation (e.g., extrahepatic neuroblastoma) but typically is insufficient for specific pediatric liver MR indications.

### Liver mass

The American College of Radiology (ACR) Pediatric Liver Imaging Reporting and Data Systems (LI-RADS) Workgroup has published consensus imaging recommendations and guidance for pediatric liver tumors, with a special focus on hepatoblastoma and hepatocellular carcinoma, that support the PRETEXT system [3]. Similar consensus imaging recommendations, built and supported by members of the Children’s Oncology Group and Society for Pediatric Radiology, have been published as well [21]. Within these recommendations, MRI has a prominent role in evaluating pediatric liver masses owing to its superior soft tissue contrast and dynamic vascular imaging capabilities.

1. Axial DWI
  a Technical considerations:
    i. FOV: Complete abdomen (lung base to iliac crest)
  b Justification: DWI/ADC reveals areas of tumor with increased cellularity and along with assessment on post-contrast imaging can help to identify areas to target if biopsy is performed. DWI in correlation with post-contrast imaging can also help identify multifocal disease within the liver, a finding of high significance when the mass is suspected to be a primary malignant tumor given the potential impact on PRETEXT and subsequent treatment decisions.
  c Motion considerations: Prone to artifacts from motion and magnetic field inhomogeneity. Navigator triggering offers superior motion compensation, resulting in higher-quality images but at the expense of longer scan times. Signal averaging is more time-efficient but is susceptible to increased motion artifacts. Newer simultaneous multi-slice (SMS) techniques provide substantial time savings and can be particularly helpful for DWI imaging [13, 22, 23].
  d Pitfalls and Pearls/Tips and Tricks: b-values of 50-100 s/mm² and 800 s/mm² generally perform well for liver mass evaluation. Lower b-values function to reveal most masses and mass components that have T2 hyperintensity while the higher B value in conjunction with ADC serves to elucidate those tumors with elements that restrict diffusion [24]. DWI can be performed prior to or following contrast injection; can fill time while waiting for the 20-minute hepatobiliary phase.
2. Axial T1 FS 3D GRE pre-contrast
  a Technical considerations:
    i. FOV: Complete abdomen (lung base to iliac crest)
  b Justification: Serves as a tool for assessment of the intrinsic T1 intensity of liver lesions, including the presence of intralesional hemorrhage, as well as a reference comparison for lesion enhancement on dynamic post-contrast imaging. This sequence may be optional when pre-contrast imaging includes Dixon imaging of the abdomen, generating a water-only image.
  c Motion considerations: Breath-hold and free-breathing sequence options are available. Breath-hold options are generally shorter sequences and may perform better in children who are able to comply with breathing instructions. Additional motion considerations addressed in dynamic post-contrast section.
  d Pitfalls and Pearls/Tips and Tricks: Pre-contrast T1 weighted sequences can be useful to identify liver lesion boundaries and interfaces with liver vasculature, helping to determine anatomic position within the liver. This sequence may also be useful for identifying multifocal disease when liver masses have signal intensity either above or below background liver parenchyma. If the breath hold is estimated to be just a little too long for the patient, the field of view can be reduced to cover the liver and kidneys.
3. Axial dynamic post-contrast
  a Overall technical considerations: Generally a 3D GRE or Dixon sequence is used.
    i. FOV: Complete abdomen (lung base to iliac crest)
    ii. Contrast: Both extracellular and hepatobiliary contrast agents can be used to evaluate liver masses in children, with the choice hinging on the suspected pathology of the mass based on prior imaging, clinical, and laboratory findings. Of the most common liver tumors encountered in children, vascular tumors are generally best evaluated with extracellular agents [25] while primary and secondary malignant liver tumors in all ages and focal nodular hyperplasia in older children and adolescents are generally better evaluated with hepatobiliary agents due to the retention of the hepatobiliary agent within functioning hepatocytes [3, 26, 27]. Two options for hepatobiliary contrast agents are available, gadoxetate disodium (Gd-EOB-DTPA, Eovist/Primovist; Bayer, Leverkusen, Germany) and gadobenate dimeglumine (Gd-BOPTA, MultiHance; Bracco Diagnostic Inc., Milan, Italy). Gadoxetate is generally preferred due to the provision of hepatobiliary phase imaging at 20 minutes compared to the 45-60 minutes needed for imaging with gadobenate. Many pediatric imaging centers administer a “double dose” (0.05 mmol/kg rather than the FDA-approved dose of 0.025 mmol/kg) of gadoxetate disodium for improved vascular imaging [3]
  b Motion considerations: Some patients who are not under anesthesia during their MRI develop transient dyspnea after intravenous injection of gadoxetate that may lead to respiratory motion resulting in degradation of image quality, especially during the arterial phase of imaging [28, 29]. Free-breathing, radial 3D GRE acquisitions (e.g., GRASP) can help improve image quality by compensating for respiratory motion. When conventional 3D GRE sequences (e.g., VIBE) are used in concert with breath-hold technique, the NSA can be increased (e.g., NSA 2) to help compensate for respiratory motion-related artifacts.
  c Pitfalls and Pearls/Tips and Tricks:
    i. Contrast timing - When using conventional 3D GRE sequences, image acquisition timing is commonly based on a standardized delay methodology where imaging for the arterial, portal venous, etc. phases commences after an appropriate time interval from the initiation of IV contrast material injection (see contrast phase timing below and Table 4). Another option is to use fluoro-triggering with the descending thoracic aorta used as a monitoring region of interest with imaging initiation triggered contrast is first visualized in the descending aorta, followed by preset delays between arterial and subsequent phases. Alternatively, sequences using free-breathing radial 3D techniques may initiate imaging prior to contrast injection and have continuous imaging after the initiation of IV contrast material administration for a defined length of time, allowing the user to set pre-determined dynamic imaging phases that are rendered available for clinical review.
    ii. Gadoxetate - Hepatic vascular assessment, an important part of the assessment when evaluating liver tumors, may be limited by the smaller dose of contrast with gadoxetate, though doubling the dose from 0.025 mmol/kg (0.1 ml/kg) to 0.05 mm/kg (0.2 ml/kg) and infusing at a slower rate can improve vascular enhancement [30, 31].
  d Axial T1 FS – Late Arterial Phase
    i. Timing: 16-20 seconds after contrast material injection
    ii. Justification: Assessment of lesion arterial phase enhancement characteristics; assessment of the hepatic arteries.
  e Axial T1 FS – Portal Venous Phase
    i. Timing: 45-60 seconds after contrast material injection
    ii. Justification: Assessment of lesion portal venous phase enhancement characteristics; assessment of the portal veins.
  f Axial T1 FS – Delayed/Transitional Phase 
    i. Timing: 2-5 minutes after contrast material injection
    ii. Justification: Assessment of lesion delayed/transitional phase enhancement characteristics; assessment of hepatic veins.
  g Axial T1 FS – Hepatobiliary Phase
    i. Timing: 20 minutes (gadoxetate), 45-60 minutes (gadobenate)
    ii. Technique: 3D GRE vs. stack of stars (StarVIBE - Siemens, LAVA Star – GE Healthcare, 3D Vane XD - Philips)
    iii. Justification: Assessment of lesion hepatobiliary phase enhancement characteristics and retention or lack thereof of the contrast agent; assessment of the biliary tree.
4. Coronal T1 FS – Hepatobiliary (Optional)
  i. Timing: 15-20 minutes (gadoxetate), 45-60 minutes (gadobenate)
  ii. Justification: Assessment of lesion hepatobiliary phase enhancement characteristics and retention or lack thereof of the contrast agent; assessment of the biliary tree.

**Table 4:** Contrast timing for liver mass evaluation

Hepatobiliary contrast for liver mass		
Mandatory sequences	Performance notes	Utility
Dynamic post-contrast		
Axial T1 FS 3D GRE – Late Arterial	16–20 s after injection	Assessment of arterial phase enhancement characteristics; assessment of hepatic artery. Note: For late arterial phase contrast should be present in the portal venous system, but not the hepatic veins
Axial T1 FS 3D GRE – Portal Venous	45–60 s after injection	Assessment of portal venous phase enhancement characteristics; assessment of portal vein
Axial T1 FS 3D GRE – Transitional	2–5 min after injection	Assessment of transitional phase enhancement characteristics; assessment of hepatic veins
Axial T1 FS 3D GRE – Hepatobiliary	15–20 min (gadoxetate) 45–60 min (gadobenate)	Assessment of hepatobiliary phase enhancement characteristics and retention or lack thereof of the contrast agent; assessment of the biliary tree
Coronal T1 FS 3D GRE – Hepatobiliary	Optional; 15–20 min	Assessment of hepatobiliary phase enhancement characteristics and retention or lack thereof of the contrast agent; assessment of the biliary tree
Extracellular contrast for liver mass		
Axial T1 FS 3D GRE – Late Arterial	16–20 s after injection	Assessment of arterial phase enhancement characteristics; assessment of hepatic artery. Note: For late arterial phase contrast should be present in the portal venous system, but not the hepatic veins
Axial T1 FS 3D GRE – Portal Venous	45–60 s after injection	Assessment of portal venous phase enhancement characteristics; assessment of portal vein
Axial T1 FS 3D GRE – Delayed	2–5 min after injection	Assessment of delayed phase enhancement characteristics; assessment of hepatic veins
Coronal T1 FS 3D GRE – Delayed	Optional; 2–5 min after injection	Assessment of delayed phase enhancement characteristics; assessment of hepatic veins

### Biliary disease/MRCP

Magnetic resonance cholangiopancreatography (MRCP) is an MR imaging technique that utilizes heavily T2-weighted sequences to highlight stationary or slow-moving fluid within the biliary tree, typically in the coronal plane. MRCP can be performed using either single-shot breath-hold or respiratory-triggered techniques [32, 33]. In recent years, there have been significant advancements in MRCP technology, including the development of newer pulse sequences and imaging protocols, which have improved image quality, reduced acquisition time, and expanded the range of indications for MRCP [34]. The application of deep learning techniques can further increase image quality [35].

1. Traditional 2D thick slab T2 SS-FSE
  a Technical considerations:
    i. FOV: Entire biliary tree
    ii. Coronal oblique acquisitions parallel to the common bile duct
  b Justification: Can be used to quickly capture clear and comprehensive overview images of the biliary tree as the thick slices allow visualization of large portions of the biliary tree within each slab.
  c Motion considerations: Typically requires breath holds so prone to motion artifact.
  d Pitfalls and Pearls/Tips and Tricks: Long readout period can result in blurring in the phase-encoding direction [36]
2. 3D-FSE MRCP 
  a Technical considerations:
    i. Typically isotropic 3D-fast spin echo (SPACE (Siemens Healthcare), Cube (GE Healthcare), and 3D GRASE or VISTA (Philips Healthcare))
    ii. FOV: Entire biliary tree
    iii. Field strength: 1.5 or 3T. Although 3T provides higher signal and smaller voxels, it is more likely to have dielectric effect artifact and susceptibility in the case of biliary stents [37, 38].
  b Justification: Compared to 3D-TSE, 3D-FSE sequences are faster and incur lower SAR. 3D sequences utilizing compressed sensing can decrease imaging time to allow for single breath-hold isovoxel acquisitions while decreasing artifact from respiratory motion and improving visualization of the bile and pancreatic ducts [39–41]. Isotropic, high-resolution images can be reformatted into different planes.
  c Motion considerations:
    i. Prone to motion artifact
  d Pitfalls and Pearls/Tips and Tricks: Respiratory triggering can prolong imaging time, but this can be mitigated with compressed sensing. It may be advantageous to obtain 3D MRCP acquisitions at the beginning of the examinations for pediatric patients when patients may be more compliant and less fatigued.
3. Hepatobiliary contrast agent-enhanced MRCP (with gadoxetate disodium, optional)
  a Technical considerations:
    i. FOV: Entire biliary tree
    ii. Typically use 3D T1 GRE sequences with fat suppression
    iii. Can be acquired in axial and/or coronal planes
    iv. Timing: 15-20 minutes following contrast injection
  b Justification: Beyond T2-weighted imaging techniques, T1-weighted sequences with hepatobiliary-specific contrast agents can be used for MRCP imaging to enhance visualization of the bile ducts and improve diagnostic accuracy. Since these contrast agents are excreted via the biliary system, they can allow for excellent visualization of the bile ducts [42].
  c Motion considerations: As discussed in the liver mass section above.
  d Pitfalls and Pearls/Tips and Tricks:
    i. Very useful to evaluate for bile leaks and to assess communication with hepatic cystic lesions
    ii. The only MRCP sequence that requires contrast administration, which prolongs imaging time
    iii. Lower image contrast and spatial resolution compared to conventional T2 MRCP

### Quantitative parenchymal characterization

Quantitative liver imaging has become routine for pediatric patients in the non-invasive evaluation of hepatic steatosis, iron overload, and fibrosis. These exams allow patients to avoid invasive biopsies to diagnose and longitudinally monitor fat, iron, and stiffness levels. Upcoming quantitative sequences (e.g., native T1, corrected T1, T1 rho, and T2 mapping) are likely to emerge as additional tools for diagnosing liver inflammation or fibrosis. However, for the purposes of these guidelines, we will review basic recommendations for performing liver iron and fat quantification and MR elastography in children. Special challenges in children include the necessity for breath-holds and inaccurate automated liver segmentation tools.

Although there are several options for the quantification of fat and iron, the most common commercially available sequence allows the simultaneous quantification of fat and iron with a single breath-hold acquisition with coverage of the entire liver. Confounder-corrected chemical shift-encoded (CSE) multi-echo spoiled gradient recalled echo (mSGRE) enables whole-liver estimation of proton density fat-fraction (PDFF) and simultaneous fat- and noise-corrected estimation of R2* (1/T2*), a quantitative biomarker of liver iron concentration (LIC) [4]. Thus, listed below are general guidelines only as standardization continues to advance.

1. Iron quantification: R2*/R2
  General: The presence of iron accelerates the decay of transverse magnetization in water protons, causing loss of signal in hepatic parenchyma in T2- and T2*-weighted imaging, which can be quantified by the transverse relaxation rates (T2 or T2*, seconds) or rates (R2 or R2*, 1/s).
    a  Technical considerations:
      i.  FOV: Entire liver
      ii.  Field strength: 1.5T or 3T. Conversion to liver iron content requires different calibration equations based on field strength. 1.5 T may be preferred in patients with known or suspected severe iron overload [43].
    b  R2* relaxometry
      i.  Justification: To determine the relaxation rate R2* of the gradient echo signal, multiple gradient-echo images are typically acquired with increasing echo times within the same repetition time (TR). Reconstruction of the R2* maps includes a fitting of signal intensity decay to estimate the R2* relaxation rate, which is highly correlated and has a linear relationship with liver iron content (LIC). In 2023, a collaborative effort by the Society for Abdominal Radiology (SAR) and the European Society for Gastrointestinal and Abdominal Radiology (ESGAR) concluded that when available, confounder-corrected R2*-based LIC quantification is the most practical method with the strongest level of evidence for accurate and reproducible quantification of LIC [43].
      ii.  Motion considerations: R2* mapping can be performed using parallel imaging to facilitate whole liver coverage in a single short breath-hold (8-12 seconds) while covering the whole liver volume in a 3D acquisition. Strategies for implementing the above sequences in a child who cannot hold their breath include increasing the signal averages and performing while free-breathing, reducing coverage to a single slice (reducing acquisition time to < 5 sec), and using respiratory navigation. New techniques utilizing 3D stack-of-stars radial acquisition [44] or multirepetition flip-angle modulated (FAM) acquisitions in combination with nonlocal means (NLM)-based motion-corrected averaging [45] show promise as free-breathing techniques for fat and iron quantification in children.
      iii.  Pitfalls and Pearls/Tips and Tricks: In patients with severe iron overload, the hepatic parenchymal signal may decay too rapidly for standard msec GRE sequences. In these patients, shortening the initial TE to less than 1 msec can be helpful.
      iv.  Post-processing: Conversion of R2* values to LIC requires field strength-specific calibration equations [46]. Although there are emerging automated segmented options, typically, regions of interest (ROIs) are drawn manually on the reconstructed R2* map to obtain R2* values. There is currently no consensus on how to draw ROIs. A common recommendation includes drawing at least 4 circular ROIs with a diameter of 2 cm or greater with at least 2 in the right lobe and at least 1 in the left lobe, avoiding areas of artifact, lesions, and vessels. The average R2* value of the 4 or more ROIs is used to estimate LIC. R2* and LIC values should be reported to the nearest integer [4].
    c  R2 relaxometry
      i.  Justification: An alternate relaxometry method for the quantification of iron is a spin-echoacquisition that uses a 90° radio frequency (RF) pulse followed by a 180° refocusing pulse and then samples the signal. The process is repeated with 5 sets of two-dimensional echo times to sample the signal decay due to the transverse relaxation and calculate the R2 relaxation rate.R2-based LIC quantification is available via the commercial products Ferriscan and Ferrismart (Resonance Health). These products are FDA-approved for LIC quantification and require an additional cost.
      ii.  Motion considerations: Images are acquired over 10-20 minutes of free breathing.
      iii.  Post-processing: For Ferriscan, acquired images are submitted electronically to Resonance Health’s data processing center for image processing, and an electronic report is returned with the mean R2 value and corresponding LIC value [47]. For Ferrismart, a cloud-based convolutional neural network trained on the database from FerriScan provides real-time analysis and instant LIC reporting [48].
2. Fat quantification
  a Technical considerations:
    i. FOV: Entire liver
    ii. Field strength: 1.5T or 3T. PDFF values are independent of field strength.
  b Justification: Chemical shift encoded MRI (CSE-MRI) computationally synthesizes T1-weighted fat-only and water-only images, from which a fat fraction (FF) map can be calculated. Confounder correction (e.g., T1 bias and T2* decay) enables whole-liver estimation of proton density fat-fraction (PDFF), a quantitative biomarker of triglyceride concentration in the liver, and has been robustly validated across field strengths and vendors.
  c Motion considerations: See above R2* section.
  d Pitfalls and Pearls/Tips and Tricks: Review the source fat and water-only images to ensure there has not been a fat/water swap. Review the auto-segmentation provided by the vendor to ensure the segmentation is accurate.
  e Post-processing: See above R2* section for ROI placement discussion. The mean PDFF is reported. Consider including the range if the fat signal is obviously heterogenous.
3. Elastography
  a Technical considerations:
    i. FOV: Typically 4 axial slices are obtained in the liver, including the widest portions and excluding the heart in the field of view.
    ii. Field strength: 1.5T and 3T. Liver stiffness is independent of field strength.
    iii. Driver set-up: The rigid passive driver is positioned over the right hepatic lobe, with the center of the paddle to the right of the nipple line. The driver should not extend to the left of the xiphoid process except in the smallest patients. Preferentially, the paddle is positioned laterally rather than medially. The goal is to position the driver over the largest portion of the liver. The paddle is fastened in place tightly with an elastic band. Driver amplitude is adjusted for patient weight [49].
    iv. Other considerations: Patients are required to fast before MRE, typically 4-6 hours.
  b Justification: The common downstream pathology of multiple pediatric liver diseases (e.g., autoimmune, metabolic, and nonalcoholic fatty liver disease) is the development of liver fibrosis and ultimately cirrhosis and end-stage liver disease. MR elastography is a technique that allows the non-invasive assessment of liver stiffness, a surrogate for hepatic fibrosis [4]. This technique has a high rate of technical success in children [49].
  c Motion considerations: Generally, acquisitions are performed with the patient at end-expiration, which ensures reproducible positioning of the diaphragm and liver [50]. However, end-inspiration breath holds can be easier for younger children and are an option. Free-breathing options are emerging [51], but not available for routine clinical use today.
  d Pitfalls and Pearls/Tips and Tricks: It is important that the paddle is tightly fastened, especially in larger patients, to ensure waves penetrate the liver. In the presence of liver iron there is a higher technical failure rate using 2D GRE-MRE compared to spin echo-echo planar imaging (SE-EPI) [49, 52]. MRE can be performed prior to or following contrast administration with no effect on liver stiffness values. For patient comfort, MRE can performed early in the exam, and if included in a full contrast liver protocol, the paddle can be removed following MRE for patient comfort for the remainder of the exam.
  e Post-processing: ROI measurements should be made by using a freehand ROI tool, sampling the largest portion of the liver on each elastogram while avoiding the peripheral 1 cm of the liver, large vessels, extrahepatic tissues, fissures, masses, and the gallbladder fossa. The arithmetic mean liver stiffness measurement is recorded in kilopascals and reported to the nearest decimal in addition to the range [4].

### Liver angiography

At times, dedicated MR angiography (MRA) might be required as part of the liver imaging protocol. Indications can be divided into two main categories: (1) congenital/portosystemic or surgical shunt evaluation and (2) vascular mapping prior to surgery. Vascular mapping could be related to the liver in general in the case of transplant and/or tumor supply/invasion in the case of resection. Inclusion of an MRA depends on institutional practices. Computed tomography angiography (CTA) with pediatric-specific protocols for dose and contrast optimization is a reasonable alternative depending on institutional experience and patient factors [53]. We focus on post-contrast MRA techniques due to their broader clinical use and more reliable vascular visualization, whereas non-contrast methods such as balanced steady-state free precession (bSSFP) and inflow-sensitive inversion recovery (IFIR) are limited by flow dependence, motion sensitivity, longer scan times, and challenges in spatial resolution, particularly in pediatric patients with altered hemodynamics [54].

1. 3D Spoiled gradient echo (post-contrast 3D MRA): GBCA
  a Technical considerations:
    i. Field of view and matrix: Coronal 3D isotropic acquisition yields the shortest scan time with best coverage for reformatting in sagittal and axial planes
    ii. Scan parameters [55]:
      1. Keep TE and TR as short as possible (TE 1-2 ms, TR 306 ms)
      2. Flip angle optimization (25-45 degrees for best signal to noise ratio (SNR))
  b Justification: Contrast-enhanced MRA is a spoiled gradient echo sequence that uses the T1 shortening properties of GBCA and flow-related vessel enhancement present at baseline to image the vasculature during and shortly after GBCA injection. Spoiler gradients accentuate T1-W and suppress background signal with greater flip angles providing greater suppression of background signal, maximizing vessel contrast. It can be acquired as a short, breath held sequence for pure arterial timing versus a longer acquisition for more detailed vessel assessment that includes both arterial and venous enhancement.
  c Motion considerations:
    i. Breath hold: If the patient is under general anesthesia, the team needs to be aware of breath holds prior to starting.
    ii. Respiratory-navigated: The target is an acceptance window of greater than 50%. Quiet free breathing is desired with shallow inspiration and prolonged expiration, to decrease diaphragm variation and prevent hyperventilating. Shorter scan times mean less bulk gross motion which may outweigh disadvantages of slight diaphragmatic blurring on angiographic imaging. Navigator placement on the spleen can be employed when imaging the liver. Pleural effusions/consolidation, asplenia or polysplenia, and iron overload can limit gating on the spleen.
  d Contrast considerations:
    i. Volume: Contrast volumes are generally low in MRI, which can be a challenge in small children and infants. To increase volume, contrast can be diluted with saline. However, it should be well mixed just before injection and typically should not be diluted by more than 50%. A double dose could be considered with attention to lifetime dose accumulation. The saline flush is important to ensure the contrast is pushed through the tubing into the patient. In general, the flush can be 10-20 ml or 2 ml/kg in patients < 10 kg. Remember, most tubing holds around 5 mL; therefore, at least 5 mL flush should be given to clear contrast from the tubing.
    ii. Rate: A rate of 1 ml/s is usually sufficient for liver angiography. Slower rates extend the contrast plateau but decrease concentration of the bolus. If the injection is too fast, the bolus might be missed. In addition, the high intravascular signal peak of a compact bolus can cause truncation (ringing) artifact, which can be confused for a filling defect. If possible, the technologist can adjust injection rate so the bolus lasts approximately half of the acquisition time. Split bolus injection is another approach, which injects half to two thirds of the contrast as a relatively fast bolus and then continues with a very slow infusion (0.5 ml/s or less) of the remainder to preserve some circulating intravascular signal throughout k-space filling.
    iii. Timing: Timing of the bolus depends on injection, patient hemodynamics, and sequence parameters. Time to acquisition can be determined with a test bolus, bolus tracking, or time-resolved imaging. A test bolus is rarely used in pediatrics because of limited contrast volume in such small patients. In bolus tracking, the full bolus is administered, sampling the artery of interest rapidly using a 2D GRE sequence that begins as contrast is injected. When the technologist sees an increase in signal intensity in the vessel of interest, they trigger the diagnostic scan when the signal threshold is reached. Because the scanner is actively scanning, it can be difficult for the patient to hear breath hold commands upon scan triggering. Steady state 3D MRA could follow a time-resolved MRA with no trigger required.
  e Pitfalls and Pearls/Tips and Tricks:
    i. Tortuous collaterals/varices can be hard to fill, which could lead to misinterpretation of thrombosis. In steady state imaging, there is loss of separation of arterial and venous phase. This could mask arterial pathology, such as splenic aneurysms, in patients with tortuous varices [56].
    ii. Generating subtraction images, maximum intensity projections (MIP), and utilizing fat suppression and T2-preparation pulses can increase conspicuity of contrast [56].
    iii. Subtraction images are often used wherein a precontrast sequence is acquired and then subtracted from post-contrast images. If there is a question of narrowing or occlusion on subtraction images, the source images should be referenced as artifact can occur from discrepancies between pre- and post-contrast images [56].
    iv. In choosing between the conventional, breath-hold triggered CE-MRA and a steady-state 3D navigated CE-MRA, the conventional method should be chosen when pure arterial imaging is required without venous contamination, the patient cannot cooperate for the time needed for navigated MRA, or there is multi-station coverage required in assessment (e.g. bolus chasing) [56].
2. 3D Spoiled gradient echo (post-contrast 3D MRA): Ferumoxytol
  a Technical considerations:
    i. Scan parameters:
      1. Flip angle decreased in comparison with GBCA 3D spoiled gradient echo
      2. Differences between 1.5T and 3T: Increase time to inversion and further decrease flip angle at 3T
    ii. Safety considerations: The FDA has issued a black box warning for Feraheme® (AMAG Pharmaceuticals, Waltham, Massachusetts) for potential acute severe hypersensitivity, identifying rapid infusion of undiluted ferumoxytol as a potential risk factor and recommending infusion over 15 minutes and hemodynamic monitoring for up to 30 minutes after infusion [57]. A multicenter registry, including 311 injections in pediatric patients < 16 years old, showed that nearly 20 years of ferumoxytol use in MRI appeared to be well tolerated, with a positive safety profile and no severe adverse events [57].
    iii. Infusion considerations: The logistics of infusion will not be discussed here, but include when (e.g. before or during scanning) and where (e.g. outside or inside scanner) ferumoxytol will be injected [58]. Ferumoxytol intravascular enhancement can last for hours to days and it can have lasting effects on imaging, including its metabolism by the reticuloendothelial system and T2* effects.
  b Justification: Ferumoxytol can be used to achieve superb, long lasting intravascular enhancement. It can be particularly useful in tortuous, dilated vessels that might have incomplete mixing or washout before uniform opacification is reached with extravascular contrast agents. In addition, the uniform opacification achieved with ferumoxytol can be useful in outlining filling defects in cases of tumor-thrombus and/or thrombosis in slow-flow, tortuous, and/or compressed vessels
  c Motion considerations: Free-breathing with respiratory navigation is most often used.
  d Pitfalls and Pearls/Tips and Tricks: Secondary to the FDA infusion recommendations, the MRA with ferumoxytol is steady state, meaning venous and arterial opacification. Because of the long intravascular half life, if a patient needs a break from scanning, they can leave the scanner and return when ready. Any GRE T1-weighted sequence can be used after ferumoxytol to assess the vascular enhancement if the MRA specific sequence is failing.
3. Time-resolved MRA
  a Technical considerations [59]:
    i. FOV: Usually a coronal acquisition
    ii. Temporal resolution: Temporal resolution is usually between 1-2 s if arterial evaluation and can be lengthened to 4-6 s if portal or systemic venous. Keyhole fraction is usually between 20-25%.
    iii. Spatial resolution: Ideal spatial resolution is between 1.0 – 1.8 mm^3
    iv. Number of dynamic phases: Usually between 8-12 if arterial focus and 10-25 if vascular malformation or venous evaluation.
  b Justification: Multiphase imaging allows for separation of arterial from venous flow and real-time visualization of flow dynamics. Time resolved MRA fills central k-space each acquisition and partially fills periphery each acquisition. The partial acquisitions of the periphery are summated to reconstruct a full volume because there should be little to no change if there isn’t significant motion. This makes it ideal for dynamic evaluation, such as in shunts and vascular malformations. Lastly, it can provide a maximal amount of arterial contrast with small bolus volumes.
  c Motion considerations: Depending on the length of scan needed, breath holding can be used. If being performed for venous or slow flow vascular malformation, the scan time usually dictates quiet free breathing.
  d Pitfalls and Pearls/Tips and Tricks: TR-MRA can be particularly useful when the contrast bolus is small. Attempts can be made to synchronize the respiratory cycle and acquisition; however, encouraging quiet free breathing might be more successful.

## Conclusion

These guidelines and suggestions represent a concerted effort from the SPR MRI and Abdominal Imaging Committees to provide expert guidance, suggestions, and rationale for the performance of core elements of pediatric liver MRI. Herein, we have strived to provide the reader with elements of and explanation for what we believe encompasses optimized pediatric liver MRI evaluation. Our hope is this document will serve as a tool for those charged with helping to care for pediatric patients with a wide variety of liver diseases where MRI may provide valuable contributions to care.

## Supplementary Information

Below is the link to the electronic supplementary material.

ESM1 (XLSX 14.3 KB)

## Data Availability

No datasets were generated or analysed during the current study.
